# *catena*-Poly[bis­(4-amino­pyridinium) [[diaqua­manganese(II)]-di-μ-chlorido] dichloride]

**DOI:** 10.1107/S1600536809026804

**Published:** 2009-07-15

**Authors:** Donia Zaouali Zgolli, Habib Boughzala, Ahmed Driss

**Affiliations:** a Laboratoire de Matériaux et Cristallochimie, Faculté des Sciences, El Manar, 2092 Tunis, Tunisia

## Abstract

Single crystals of the title organic–inorganic hybrid, {(C_5_H_7_N_2_)_2_[MnCl_2_(H_2_O)_2_]Cl_2_}_*n*_, were synthesized from an ethanol solution containing manganese(II) chloride tetra­hydrate and 4-amino­pyridine under acidic conditions. The asymmetric unit contains a disordered organic cation (occupancies in the ratio 0.72:0.28), a chloride anion and an MnCl(H_2_O) moiety with the Mn^II^ atom located on an inversion center. The structure is built up of infinite chains of edge-sharing [MnCl_4_(H_2_O)_2_] octa­hedra developing parallel to the *a* axis which are separated by the 4-amino­pyridinium ions and discrete chloride ions. The organic cations occupy the empty space around each inorganic chain. Structural cohesion is organized through N—H⋯Cl and O—H⋯Cl hydrogen bonds, which build up a three-dimensional network.

## Related literature

For general background to organic–inorganic hybride materials, see: Lacroix *et al.* (1994 ▶); Mitzi (2001 ▶); Calabrese *et al.* (1991 ▶); Hong *et al.* (1992 ▶). For related structures, see: Caputo *et al.* (1976 ▶); Hachuła *et al.* (2009 ▶); Zeng *et al.* (2008 ▶).
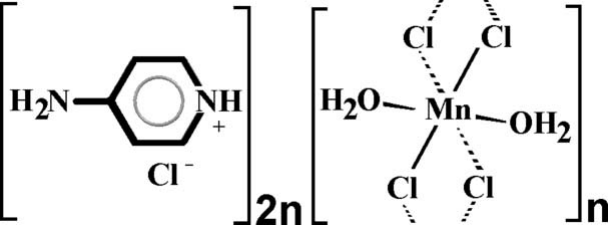


## Experimental

### 

#### Crystal data

(C_5_H_7_N_2_)_2_[MnCl_2_(H_2_O)_2_]Cl_2_
*M* *_r_* = 421.01
Monoclinic, 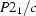

*a* = 3.946 (1) Å
*b* = 17.586 (6) Å
*c* = 12.845 (4) Å
β = 93.48 (3)°
*V* = 889.7 (5) Å^3^
*Z* = 2
Mo *K*α radiation
μ = 1.35 mm^−1^
*T* = 298 K
0.05 × 0.04 × 0.02 mm

#### Data collection

Enraf–Nonius CAD-4 diffractometer
Absorption correction: ψ scan (North *et al.*, 1968 ▶) *T*_min_ = 0.916, *T*_max_ = 0.999
2516 measured reflections
1892 independent reflections
1473 reflections with *I* > 2σ(*I*)
*R*_int_ = 0.017
2 standard reflections frequency: 120 min intensity decay: 1%

#### Refinement

*R*[*F* ^2^ > 2σ(*F* ^2^)] = 0.036
*wR*(*F* ^2^) = 0.095
*S* = 1.04
1892 reflections
112 parameters
43 restraints
H-atom parameters constrained
Δρ_max_ = 0.38 e Å^−3^
Δρ_min_ = −0.45 e Å^−3^

### 

Data collection: *CAD-4 EXPRESS* (Duisenberg, 1992 ▶; Macíček & Yordanov, 1992 ▶); cell refinement: *CAD-4 EXPRESS*; data reduction: *XCAD4* (Harms & Wocadlo, 1995 ▶); program(s) used to solve structure: *SHELXS97* (Sheldrick, 2008 ▶); program(s) used to refine structure: *SHELXL97* (Sheldrick, 2008 ▶); molecular graphics: *PLATON* (Spek, 2009 ▶) and *DIAMOND* (Brandenburg, 2006 ▶); software used to prepare material for publication: *WinGX* (Farrugia, 1999 ▶).

## Supplementary Material

Crystal structure: contains datablocks I, global. DOI: 10.1107/S1600536809026804/dn2460sup1.cif

Structure factors: contains datablocks I. DOI: 10.1107/S1600536809026804/dn2460Isup2.hkl

Additional supplementary materials:  crystallographic information; 3D view; checkCIF report

## Figures and Tables

**Table 1:** Hydrogen-bond geometry (Å, °)

*D*—H⋯*A*	*D*—H	H⋯*A*	*D*⋯*A*	*D*—H⋯*A*
O1—H*W*1⋯Cl1^i^	0.87	2.27	3.090 (2)	158
O1—H*W*2⋯Cl1^ii^	0.73	2.39	3.082 (2)	159
N1—H1*A*⋯Cl1	0.86	2.41	3.264 (4)	172
N1—H1*B*⋯Cl2	0.86	2.57	3.415 (4)	169
N1′—H1′1⋯Cl1^iii^	0.86	2.47	3.299 (10)	163
N1′—H1′2⋯Cl1^i^	0.86	2.58	3.386 (9)	156

Symmetry codes: (i) 
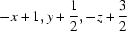
; (ii) 
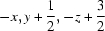
; (iii) 
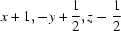
.

**Fig. 1.**
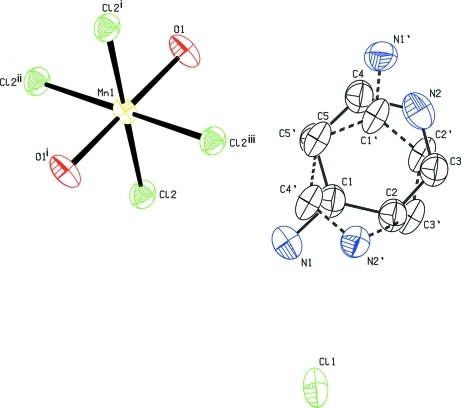
Representation of the assymetric unit with the atom labelling scheme. Displacement ellipsoids are drawn at the 50% probability level. H atoms ahve been omitted for clarity. [Symmetry codes: (i) -x + 1, -y + 1, -z + 2; (ii) -x, -y + 1, -z + 2; (iii) x + 1, y, z; (iv) x - 1, y, z]

**Fig. 2.**
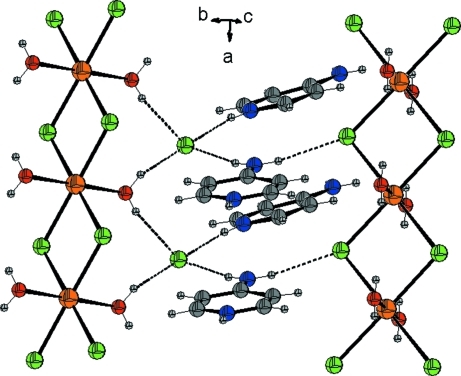
Partial packing view showing the hydrogen bond interactions between the inorganic and organic molecules. Ellipsoids are drawn at the 50% probability level. Hydrogen bonds are shown as dashed lines.

**Fig. 3.**
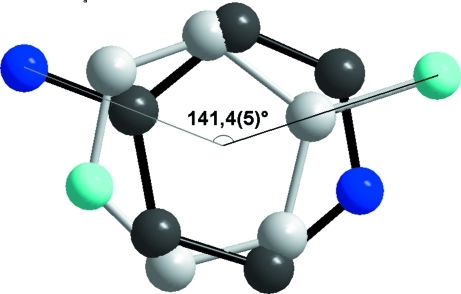
Representation of the disordrered components of the organic cation

## supplementary crystallographic information

### Comment

Studies of organic-inorganic hybrid compounds continue to be a focus area in chemistry and material science because they combine properties of organic and inorganic compounds within one single molecular scale, such as second order nonlinear optical (NLO) response, magnetism, luminescence, and even multifunctional properties (Mitzi *et al.* (2001); Lacroix *et al.* (1994)).

This kind of materials, generally expressed as (R—NH_3_)_2_-*MX*_4_ or (NH_3_– R—NH_3_) *MX*_4_ (where *R*: organic group, *M*: divalent metal and *X*: halogen) can be regarded as semiconductor/insulator multiple quantum well system consisting of metal halide semiconductor layers sandwiched between organic ammonium insulator layers (Calabrese *et al.* (1991); Hong *et al.* (1992). In this paper, we report the synthesis and single-crystal X-ray diffraction studies of the organic-inorganic hybrid compound: [MnCl_2_(H_2_O)_2_].(C_5_H_8_N_2_)_2_.Cl_2_.

The assymetric unit is built up from a MnCl(H_2_O) moiety, a fully disordered 4-ammoniumpyridine and a Cl ion. The Mn atom is located on an inversion center and each managanese atom is octahedrally coordinated to four equatorial chlorine atoms and to two oxygen atoms in axial positions (Fig. 1).

The [MnCl_2_(H_2_O)_2_].(C_5_H_8_N_2_)_2_.Cl_2 _structure is built up of infinite edges sharing octahedra MnCl_4_(H_2_O)_2_ chains running along the [100] direction. Similar arrangement of the inorganic part was reported for [(CH_3_)_3_NH]MnCl_3_. 2H_2_O published by Caputo *et al.* (1976). The organic-inorganic cohesion is ensured by hydrogen bonding that involves two kinds of interactions: N1—H1A···Cl1 and N1—H1B···Cl2 bonds between the organic cation and the chloride and O—HW1···Cl1 and O—HW2···Cl1 between the water molecule and the Cl anion (Fig. 2, Table 1). It is worthy to note that the second kind of hydrogene bonds are stronger than the first one.

The [C_5_H_8_N_2_]^+^cations is disordered over two positions which are rotated with respect to each other by about 141°. Thus, the amine group of one component lies close to the carbon atom C1 of the other component so that both components are more or less coplanar one to another (Fig. 3).

The distances and angles througout the structure are in good agreement with those encountered in several compounds of literature (Zeng *et al.* (2008); Hachuła *et al.* (2009)).

### Experimental

An aqueous HCl (1*M*) solution, 4-aminopyridine (C_5_H_6_N_2_) and manganese dichloride tetrahydrate (MnCl_2_.4H_2_O) in a 2:1:1 molar ratio were mixed and dissolved in sufficient ethanol. Crystal for X-Ray diffraction structural analysis were grown by slow evaporation at room temperature and then set aside for few days to obtain colourless crystals.

### Refinement

All H atoms attached to C atoms and N atom were fixed geometrically and treated as riding with C—H = 0.93 Å and N—H = 0.86 Å with *U*_iso_(H) = 1.2*U*_eq_(C or N). H atoms of water molecule were located in difference Fourier maps and included in the subsequent refinement using restraints (O—H= 0.82 (1)Å and H···.H= 1.39 (2) Å) with *U*_iso_(H) = 1.5*U*_eq_(O). In the last stage of refinement, they were treated as as riding on the O atom.

The organic cation is disordered over two positions twisted to each other by about 141° around an axis perpendicular to their mean planes. The two components were refined using the tools available in *SHELXL97*(Sheldrick, 2008): PART, SAME and EADP. In the first step of refinement the occupancy factor for each domain has been determined to be in the ration 0.72/0.28 by using the FREE variable option.

### Crystal data

**Table:**

(C_5_H_7_N_2_)_2_[MnCl_2_(H_2_O)_2_]Cl_2_	*F*(000) = 426
*M**_r_* = 421.01	*D*_x_ = 1.572 Mg m^−^^3^
Monoclinic, *P*2_1_/*c*	Mo *K*α radiation, λ = 0.71073 Å
Hall symbol: -P 2ybc	Cell parameters from 25 reflections
*a* = 3.946 (1) Å	θ = 10–15°
*b* = 17.586 (6) Å	µ = 1.35 mm^−^^1^
*c* = 12.845 (4) Å	*T* = 298 K
β = 93.48 (3)°	Prism, colourless
*V* = 889.7 (5) Å^3^	0.05 × 0.04 × 0.02 mm
*Z* = 2	

### Data collection

**Table:**

Enraf–Nonius CAD-4 diffractometer	1473 reflections with *I* > 2σ(*I*)
Radiation source: fine-focus sealed tube	*R*_int_ = 0.017
graphite	θ_max_ = 27.0°, θ_min_ = 2.3°
non–profiled ω/2θ scans	*h* = −5→1
Absorption correction: ψ scan (North *et al.*, 1968)	*k* = 0→22
*T*_min_ = 0.916, *T*_max_ = 0.999	*l* = −16→16
2516 measured reflections	2 standard reflections every 120 min
1892 independent reflections	intensity decay: 1%

### Refinement

**Table:**

Refinement on *F*^2^	Primary atom site location: structure-invariant direct methods
Least-squares matrix: full	Secondary atom site location: difference Fourier map
*R*[*F*^2^ > 2σ(*F*^2^)] = 0.036	Hydrogen site location: inferred from neighbouring sites
*wR*(*F*^2^) = 0.095	H-atom parameters constrained
*S* = 1.04	*w* = 1/[σ^2^(*F*_o_^2^) + (0.0506*P*)^2^ + 0.1303*P*] where *P* = (*F*_o_^2^ + 2*F*_c_^2^)/3
1892 reflections	(Δ/σ)_max_ = 0.001
112 parameters	Δρ_max_ = 0.38 e Å^−^^3^
43 restraints	Δρ_min_ = −0.45 e Å^−^^3^

### Special details

**Table:**

Experimental. Refinement of F^2^ against ALL reflections. The weighted *R*-factor *wR* and goodness of fit S are based on F^2^, conventional *R*-factors *R* are based on F, with F set to zero for negative F^2^. The threshold expression of F^2^ > 2sigma(F^2^) is used only for calculating *R*-factors(gt) *etc*. and is not relevant to the choice of reflections for refinement. *R*-factors based on F^2^ are statistically about twice as large as those based on F, and R– factors based on ALL data will be even larger.
Geometry. All e.s.d.'s (except the e.s.d. in the dihedral angle between two l.s. planes) are estimated using the full covariance matrix. The cell e.s.d.'s are taken into account individually in the estimation of e.s.d.'s in distances, angles and torsion angles; correlations between e.s.d.'s in cell parameters are only used when they are defined by crystal symmetry. An approximate (isotropic) treatment of cell e.s.d.'s is used for estimating e.s.d.'s involving l.s. planes.
Refinement. Refinement of *F*^2^ against ALL reflections. The weighted *R*-factor *wR* and goodness of fit *S* are based on *F*^2^, conventional *R*-factors *R* are based on *F*, with *F* set to zero for negative *F*^2^. The threshold expression of *F*^2^ > σ(*F*^2^) is used only for calculating *R*-factors(gt) *etc*. and is not relevant to the choice of reflections for refinement. *R*-factors based on *F*^2^ are statistically about twice as large as those based on *F*, and *R*- factors based on ALL data will be even larger.

### Fractional atomic coordinates and isotropic or equivalent isotropic displacement parameters (Å^2^)

**Table:**

	*x*	*y*	*z*	*U*_iso_*/*U*_eq_	Occ. (<1)
Mn1	0.5000	0.5000	1.0000	0.03113 (15)	
Cl1	0.05805 (18)	0.09651 (5)	0.82588 (5)	0.0535 (2)	
Cl2	−0.01007 (15)	0.40847 (3)	0.95374 (5)	0.03833 (17)	
O1	0.4739 (4)	0.54280 (11)	0.84390 (13)	0.0451 (5)	
HW1	0.6426	0.5601	0.8110	0.068*	
HW2	0.3186	0.5559	0.8160	0.068*	
N1	0.2765 (11)	0.2731 (2)	0.7899 (3)	0.0640 (10)	0.72
H1A	0.2411	0.2253	0.7984	0.077*	0.72
H1B	0.2264	0.3050	0.8374	0.077*	0.72
N2	0.6831 (9)	0.3534 (2)	0.5246 (3)	0.0569 (9)	0.72
C1	0.4091 (13)	0.2978 (3)	0.7031 (3)	0.0501 (6)	0.72
C2	0.4949 (15)	0.2487 (3)	0.6239 (4)	0.0501 (6)	0.72
H2	0.4608	0.1966	0.6298	0.060*	0.72
C3	0.6292 (14)	0.2782 (3)	0.5378 (4)	0.0501 (6)	0.72
H3	0.6869	0.2451	0.4853	0.060*	0.72
C4	0.6020 (12)	0.4012 (3)	0.6012 (3)	0.0501 (6)	0.72
H4	0.6453	0.4528	0.5937	0.060*	0.72
C5	0.4562 (16)	0.3764 (4)	0.6909 (5)	0.0501 (6)	0.72
H5	0.3920	0.4107	0.7412	0.060*	0.72
N1'	0.704 (3)	0.4342 (5)	0.5481 (7)	0.060 (3)	0.28
H1'1	0.7695	0.4343	0.4855	0.072*	0.28
H1'2	0.6962	0.4761	0.5824	0.072*	0.28
N2'	0.400 (2)	0.2381 (5)	0.6940 (6)	0.049 (2)	0.28
C1'	0.614 (3)	0.3697 (5)	0.5917 (8)	0.0483 (15)	0.28
C2'	0.619 (4)	0.3023 (5)	0.5432 (10)	0.0483 (15)	0.28
H2'	0.6893	0.2985	0.4756	0.058*	0.28
C3'	0.517 (4)	0.2379 (6)	0.5971 (8)	0.0483 (15)	0.28
H3'	0.5299	0.1912	0.5635	0.058*	0.28
C4'	0.377 (3)	0.3078 (5)	0.7366 (9)	0.0483 (15)	0.28
H4'	0.2683	0.3122	0.7986	0.058*	0.28
C5'	0.507 (4)	0.3743 (10)	0.6929 (12)	0.0483 (15)	0.28
H5'	0.5207	0.4197	0.7301	0.058*	0.28

### Atomic displacement parameters (Å^2^)

**Table:**

	*U*^11^	*U*^22^	*U*^33^	*U*^12^	*U*^13^	*U*^23^
Mn1	0.0294 (3)	0.0381 (3)	0.0263 (2)	0.0004 (2)	0.00499 (18)	0.00265 (19)
Cl1	0.0426 (4)	0.0783 (5)	0.0402 (3)	−0.0008 (3)	0.0075 (3)	−0.0189 (3)
Cl2	0.0326 (3)	0.0375 (3)	0.0455 (3)	0.0002 (2)	0.0071 (2)	−0.0063 (2)
O1	0.0335 (9)	0.0706 (13)	0.0317 (9)	0.0022 (9)	0.0050 (7)	0.0172 (8)
N1	0.086 (3)	0.061 (2)	0.0471 (19)	−0.003 (2)	0.0207 (19)	0.0011 (17)
N2	0.051 (2)	0.078 (3)	0.0413 (19)	0.0024 (19)	0.0022 (16)	0.0057 (18)
C1	0.0557 (14)	0.0510 (11)	0.0435 (11)	0.0032 (12)	0.0032 (10)	−0.0060 (10)
C2	0.0557 (14)	0.0510 (11)	0.0435 (11)	0.0032 (12)	0.0032 (10)	−0.0060 (10)
C3	0.0557 (14)	0.0510 (11)	0.0435 (11)	0.0032 (12)	0.0032 (10)	−0.0060 (10)
C4	0.0557 (14)	0.0510 (11)	0.0435 (11)	0.0032 (12)	0.0032 (10)	−0.0060 (10)
C5	0.0557 (14)	0.0510 (11)	0.0435 (11)	0.0032 (12)	0.0032 (10)	−0.0060 (10)
N1'	0.094 (8)	0.049 (5)	0.038 (4)	−0.017 (5)	0.002 (5)	−0.003 (4)
N2'	0.052 (5)	0.050 (5)	0.045 (5)	0.000 (4)	0.005 (4)	−0.004 (4)
C1'	0.052 (3)	0.040 (3)	0.052 (3)	0.008 (2)	−0.002 (3)	−0.018 (3)
C2'	0.052 (3)	0.040 (3)	0.052 (3)	0.008 (2)	−0.002 (3)	−0.018 (3)
C3'	0.052 (3)	0.040 (3)	0.052 (3)	0.008 (2)	−0.002 (3)	−0.018 (3)
C4'	0.052 (3)	0.040 (3)	0.052 (3)	0.008 (2)	−0.002 (3)	−0.018 (3)
C5'	0.052 (3)	0.040 (3)	0.052 (3)	0.008 (2)	−0.002 (3)	−0.018 (3)

### Geometric parameters (Å, °)

**Table:**

Mn1—O1^i^	2.1383 (17)	C3—H3	0.9300
Mn1—O1	2.1383 (17)	C4—C5	1.389 (7)
Mn1—Cl2^ii^	2.6117 (8)	C4—H4	0.9300
Mn1—Cl2^iii^	2.6117 (8)	C5—H5	0.9300
Mn1—Cl2	2.6173 (8)	N1'—C1'	1.323 (11)
Mn1—Cl2^i^	2.6173 (8)	N1'—H1'1	0.8600
Cl2—Mn1^iv^	2.6117 (8)	N1'—H1'2	0.8600
O1—HW1	0.8654	N2'—C4'	1.348 (10)
O1—HW2	0.7285	N2'—C3'	1.353 (10)
N1—C1	1.332 (5)	C1'—C2'	1.339 (11)
N1—H1A	0.8600	C1'—C5'	1.393 (13)
N1—H1B	0.8600	C2'—C3'	1.400 (12)
N2—C4	1.348 (5)	C2'—H2'	0.9300
N2—C3	1.351 (6)	C3'—H3'	0.9300
C1—C2	1.392 (6)	C4'—C5'	1.408 (13)
C1—C5	1.405 (8)	C4'—H4'	0.9300
C2—C3	1.359 (6)	C5'—H5'	0.9300
C2—H2	0.9300		
			
O1^i^—Mn1—O1	180.000 (1)	N2—C3—C2	123.2 (4)
O1^i^—Mn1—Cl2^ii^	90.13 (6)	N2—C3—H3	118.4
O1—Mn1—Cl2^ii^	89.87 (6)	C2—C3—H3	118.4
O1^i^—Mn1—Cl2^iii^	89.87 (6)	N2—C4—C5	122.6 (5)
O1—Mn1—Cl2^iii^	90.13 (6)	N2—C4—H4	118.7
Cl2^ii^—Mn1—Cl2^iii^	180.0	C5—C4—H4	118.7
O1^i^—Mn1—Cl2	89.37 (6)	C4—C5—C1	117.8 (6)
O1—Mn1—Cl2	90.63 (6)	C4—C5—H5	121.1
Cl2^ii^—Mn1—Cl2	97.99 (3)	C1—C5—H5	121.1
Cl2^iii^—Mn1—Cl2	82.01 (3)	C1'—N1'—H1'1	120.0
O1^i^—Mn1—Cl2^i^	90.63 (6)	C1'—N1'—H1'2	120.0
O1—Mn1—Cl2^i^	89.37 (6)	H1'1—N1'—H1'2	120.0
Cl2^ii^—Mn1—Cl2^i^	82.01 (3)	C4'—N2'—C3'	114.4 (10)
Cl2^iii^—Mn1—Cl2^i^	97.99 (3)	N1'—C1'—C2'	123.4 (12)
Cl2—Mn1—Cl2^i^	180.00 (2)	N1'—C1'—C5'	116.6 (10)
Mn1^iv^—Cl2—Mn1	97.99 (3)	C2'—C1'—C5'	120.0 (13)
Mn1—O1—HW1	126.0	C1'—C2'—C3'	118.3 (12)
Mn1—O1—HW2	124.3	C1'—C2'—H2'	120.9
HW1—O1—HW2	107.3	C3'—C2'—H2'	120.9
C1—N1—H1A	120.0	N2'—C3'—C2'	125.2 (10)
C1—N1—H1B	120.0	N2'—C3'—H3'	117.4
H1A—N1—H1B	120.0	C2'—C3'—H3'	117.4
C4—N2—C3	118.2 (4)	N2'—C4'—C5'	123.9 (13)
N1—C1—C2	122.3 (5)	N2'—C4'—H4'	118.0
N1—C1—C5	118.4 (5)	C5'—C4'—H4'	118.0
C2—C1—C5	119.3 (5)	C1'—C5'—C4'	117.5 (14)
C3—C2—C1	118.8 (5)	C1'—C5'—H5'	121.3
C3—C2—H2	120.6	C4'—C5'—H5'	121.3
C1—C2—H2	120.6		

Symmetry codes: (i) −*x*+1, −*y*+1, −*z*+2; (ii) *x*+1, *y*, *z*; (iii) −*x*, −*y*+1, −*z*+2; (iv) *x*−1, *y*, *z*.

### Hydrogen-bond geometry (Å, °)

**Table:**

*D*—H···*A*	*D*—H	H···*A*	*D*···*A*	*D*—H···*A*
O1—HW1···Cl1^v^	0.87	2.27	3.090 (2)	158
O1—HW2···Cl1^vi^	0.73	2.39	3.082 (2)	159
N1—H1A···Cl1	0.86	2.41	3.264 (4)	172
N1—H1B···Cl2	0.86	2.57	3.415 (4)	169
N1'—H1'1···Cl1^vii^	0.86	2.47	3.299 (10)	163
N1'—H1'2···Cl1^v^	0.86	2.58	3.386 (9)	156

Symmetry codes: (v) −*x*+1, *y*+1/2, −*z*+3/2; (vi) −*x*, *y*+1/2, −*z*+3/2; (vii) *x*+1, −*y*+1/2, *z*−1/2.
